# Statistical modelling for optimized lyophilization of *Lactobacillus acidophilus* strains for improved viability and stability using response surface methodology

**DOI:** 10.1186/s13568-018-0659-3

**Published:** 2018-08-10

**Authors:** Lakshminarayana Turuvekere Sadguruprasad, Madhusudhan Basavaraj

**Affiliations:** 1 Research Center for Nanoscience and Technology, Department of Studies and Research in Biochemistry, Bio-Sciences Block, Davangere University, Shivagangotri, Davanagere, Karnataka 577002 India; 2 Research Center for Nanoscience and Technology, Department of Studies and Research in Biochemistry & Food Technology, Bio-Sciences Block, Davangere University, Shivagangotri, Davanagere, Karnataka 577002 India

**Keywords:** *L. acidophilus*, Lyoprotectors, Suspension media, Survival rate, Storage

## Abstract

The awareness regarding use of probiotics for health benefits has created a huge market worldwide. The use of probiotic cultures and its success in commercial scale is relying on the techniques employed to ensure that the potential cultures with desired functional activity are stable and viable for longer period. The present study was undertaken to statistically evaluate the effect of lyoprotectant in the suspension media containing sucrose and reconstituted skim milk on the viability of *Lactobacillus acidophilus* strains. The central composite rotatable design of response surface methodology was used in evaluating the survival rate and viability even after 24 months’ storage of three different lyophilized strains of *L. acidophilus*. We also determined the exclusive use of lyoprotectant on the survival of the lyophilized strains throughout the study period without altering any of the culture conditions. The most conspicuous result to emerge from the data is that the suspension medium composed of minimal amount of sucrose (~ 1.2%) in the reconstituted skim milk (6%) was evident in maintaining a higher survival rate of lyophilized culture during 2-year storage. From this investigation, the optimized suspension media used to obtain the lyophilized concentrate of *L. acidophilus* could be recommended in designing dietary supplement with minimal modification for industrial application. This might reduce the manufacturing cost as well as storage loss occurring both in pharma and food sector.

**Electronic supplementary material:**

The online version of this article (10.1186/s13568-018-0659-3) contains supplementary material, which is available to authorized users.

## Introduction

Probiotics are viable microorganisms gaining importance for optimum health benefits to alleviate the gut related disorders and modulate immune response (Morelli and Capurso 2012). Generally, commercialized probiotic products invariably consist of *Lactobacilli*, *Bifidobacteria* and also some yeasts for positive outcomes (Devi et al. 2015; Hudson et al. 2016; Shokri et al. 2015). The probiotic activity is shown by inhibiting the adherence of previously harbored bacteria, changing luminal pH, and reinforcing the body’s natural defense by producing antibacterial peptides (Marchesi et al. 2015). In recent days, some clinical practitioners have begun to use probiotics for the treatment of many diseases (Hickson 2013; Hungin et al. 2013). The comparable outcome of the data between the in vitro models and in vivo human trials has substantiated that more attention on the strain specificity, therapeutic potential and promising methodologies in culturing the strains has to be paid to meet the commercial demand (Shanahan et al. 2012). The international scientific association for probiotics and prebiotics, states that any commercialized product or even marketable probiotic product must contain at least 10^6^–10^7^ CFU (colony forming unit) of viable probiotic per gram of the product during the time of consumption to exert probiotic effects on human health (Hill et al. 2014).

Unfortunately, the viability problem crops-up during storage of the product. Therefore, the probiotics enriched products for commercial supply needs to be developed to meet the drawbacks in storage in all kinds of weather. Earlier researchers have emphasized the use of optimal preservation methods to be employed during long-term storage for the efficacy of probiotic products with higher viability of the microorganisms (Bigliardi and Galati 2013). Moreover, investigators have successfully attempted both lyophilization and spray-drying methods to dehydrate some selected probiotic cultures while exposing the culture to extreme environmental conditions (Martín et al. 2015). The simplest classical method, lyophilization has been widely used for the past several years to produce viable culture in dried powder form. However, the adverse effect of the process on the viability of many microorganisms is still inconclusive (Kandil and El Soda 2015). Apart from that recently, studies concerning to preparation of concentrated starter culture of well-known microorganisms by lyophilization was tried out for food application (Lee et al. 2016). The process has beneficial effects on the dried organisms as the entire process works at frozen temperature with no thermal degradation (Jalali et al. 2012). Earlier researchers have vigorously tested lyoprotective agents in order to improve the survival of bacteria during preservation (Lee et al. 2016). The continued efforts of the investigators began to add information that was much needed at the time of optimization considering the stability of microorganisms during the lyophilization process and long-term storage (She and Petti 2015).

Several researchers have tried to develop such a probiotic product in a cost-effective manner with high CFU. Regrettably, apart from few researchers, least attention has been paid to address the loss of crucial characteristics include sustainable probiotic activity, flavor, viable cells count, shelf-life on prolonged storage for gastrointestinal tract application (De Prisco and Mauriello 2016). Improvement of the process was continuous and few investigators have attempted to present the data using scalable statistical models to describe survival curves of well-considered microorganisms for post-lyophilized storage (Miyamoto-Shinohara et al. 2008; Silva et al. 2009). Such statistically definable models would allow the systematic analysis of any experimental data by using limited parameters of interest that would correlate between the parameters used with survival rates of microorganisms.

To overcome some of these gaps, we have employed central composite rotatable design (CCRD) of response surface methodology (RSM) as a tool to objectively evaluate the survival behavior of lyophilized microorganisms for 24-month storage.

## Materials and methods

### Materials

Lactobacillus MRS broth (M369), Lactobacillus MRS agar (M641), sucrose (GRM601), skim milk (M530), l-cysteine hydrochloride (TC058), phosphate buffered saline (M1866) were purchased from HiMedia Laboratories Pvt. Ltd. (Mumbai). Water used for all experiments was Milli-Q water. All other chemicals were of highest purity available and used without further purification.

### Microorganisms and culture conditions

*Lactobacillus acidophilus* (*NCDC 291*), *L. acidophilus* (*NCDC 015*) and *L. acidophilus* (*MTCC 10307*) were procured from National Collection of Dairy Cultures (ICAR-NDRI, Karnal, India) and Microbial Type Culture Collection and Gene Bank (Institute of Microbial Technology, Chandigarh, India), respectively. The bacteria were kept at − 20 °C until used. All 3 strains were subcultured at least twice in MRS medias at 37 °C just prior to experimental use.

### Experimental design and its statistical modeling

Based on the previous studies, we have chosen sucrose and reconstituted skim milk as recommended lyoprotectants for lyophilization. We have adopted statistical modelling-an applied mathematical technique to optimize process by considering the affecting factors to achieve maximum response with relative significance. Thus, the five-level-two-factor CCRD of RSM (Kim and Rhee 2015) was used to optimize experimental design and the data is shown in Table 1. The design includes 13 experimental runs consisting of 4 factorial points, wherein the factors used in full factorial runs are studied at + 1 and − 1 levels. Similarly, 4 axial points responsible to make design rotatable (Santos et al. 2015) and 5 center points involving all factors centered to help in determining the curvature and replication for estimating pure error (Additional file 1: Tables S1, S2, Figure S1). Verifiable models depicting the experimental results were developed using the data from the designed experiments. The model parameters were estimated using a factorial model for two factor (except survival rate which was linear model), each at level is as shown below

1 $$Y = \beta_{0} + \beta_{1} X_{1} + \beta_{2} X_{2} + \beta_{12} X_{1} X_{2}$$

where Y is the expected value of the response variables, and β_0_, β_1_, β_2_ and β_12_ are the model parameters. The X_1_, X_2_ and X_12_ are the coded factors evaluated. In this study, concentration of sucrose and reconstituted skim milk were selected as main factors. Initially only *L. acidophilus* (NCDC 291) culture was used for conducting the experiments and all the three *L. acidophilus* cultures (*NCDC 291*, *NCDC 015* and *MTCC 10307*) separately were tested during optimization.

**Table 1:** The coded levels of sucrose and reconstituted skim milk for central composite experimental design

Factors [% (g/100 mL)]	Coded levels				
	− alpha	Low	Center	High	+ alpha
Sucrose (S)	− 1.24264	0	3	6	7.24264
Reconstituted skim milk (SM)	− 1.24264	0	3	6	7.24264

The statistical software package Design-Expert^®^ (Version 7.0.0, Stat-Ease, Inc., Minneapolis, MN, USA) was used for analysis of the experimental data, to yield regression equation and determine optimum parameters by the plot of response contours and surface graphs. The response values were fitted to linear model for survival rate and a two-factorial interaction (2FI) model for rest of the responses after evaluating the results by Design-Expert^®^ software.

### Lyophilization

The bacterial cultures were collected from previously subcultured strains and inoculated into MRS media. The individual culture medium was incubated at 37 °C for 24 h. The cells were harvested at stationery growth phase by centrifuging at 5000 RPM for 10 min at 4 °C. The cells were washed twice by phosphate buffered saline and centrifuged. The washed cells were resuspended in suspension media containing reconstituted skim milk (SM) and sucrose (S) as lyoprotectors in recommended ratios by statistical model. However, in two runs negative concentration was suggested, however as its impossible to have negative concentration we decided to consider it as 0 concentration. The resultant homogeneous suspension was re-suspended in aliquots (25 mL) of each bacterial strain. Samples (1–2 mL) were taken from each aliquot and the number of CFU/mL was determined by the plate dilution method using MRS media. Immediately, the aliquots were frozen in sterile cryovials at − 40 °C and stored for 24 h. The frozen samples were dehydrated using lyophilizer (Labconco Corporation, USA) under high vacuum (0.05 mbar) for 48 h. Lyophilized cells were stored in tightly sealed cryovials at different temperatures (− 80, − 40, − 20, 4 °C and at room temperature) in the dark for up to 24 months to assess the stability (Shu et al. 2018). We have presented the data for samples processed at 4 °C (and the data on other temperatures has not been presented here).

### Cell viability assay

Prior to the assay, lyophilized samples were added with suspension media by maintaining the original ratio and allowed for 10 min at room temperature for rehydration. Then, cells were diluted serially in MRS media and further incubated at 37 °C for 24 h and the number of CFU/mL was calculated (Qi et al. 2017; Shu et al. 2017). The procedure was repeated for the lyophilized samples in every 6 months until 2-year storage period. The survival rates were calculated and tabulated in Table 2 as follows:

$${\text{Survival}}\;{\text{Rate}}\,(\%) = \frac{{\text{Viable count after lyophilization (}VCAL)\;{\text{in}}\;{\text{CFU/mL}}}}{{\text{Viable count before lyophilization (}VCBL\text{)}\;{\text{in}}\;{\text{CFU/mL}}}}*100$$

**Table 2:** Survival rates of *L. acidophilus* before and after lyophilization in 13 different formulations

Runs	Sucrose	Skim milk	VCBL	VCAL	Survival rate
	% (g/100 mL)		CFU/mL		%
1	0	0	1.98 × 10^5^	9.91 × 10^4^	50.03
2	6	0	9.46 × 10^11^	5.92 × 10^11^	62.61
3	0	6	8.90 × 10^11^	6.02 × 10^11^	67.63
4	6	6	8.05 × 10^10^	6.13 × 10^10^	76.19
5	− 1.24^a^	3	6.10 × 10^8^	3.67 × 10^8^	60.21
6	7.24	3	1.63 × 10^11^	1.12 × 10^11^	68.83
7	3	− 1.24^a^	6.02 × 10^8^	3.47 × 10^8^	57.62
8	3	7.24	2.87 × 10^11^	2.15 × 10^11^	74.86
9	3	3	1.73 × 10^10^	1.14 × 10^10^	65.96
10	3	3	1.73 × 10^10^	1.14 × 10^10^	65.73
11	3	3	1.73 × 10^10^	1.14 × 10^10^	65.97
12	3	3	1.74 × 10^10^	1.14 × 10^10^	65.72
13	3	3	1.74 × 10^10^	1.14 × 10^10^	65.45

^a^As negative concentration cannot be achieved 0 was considered

## Results

The cell viability assessment is an important criterion in any microbial based pharma and food products of commercial interests. Till date, several studies have been undertaken by the researchers to improve the cell viability in a set of environmental conditions congenial for human use. In this investigation, we have chosen sucrose and reconstituted skim milk as recommended lyoprotectants prior to lyophilization followed by statistical modelling to optimize the process to achieve maximum response with relative significance. In the experimental design, thirteen runs were designed using CCRD. The design matrix and the corresponding results of CCRD experiments to determine the effects of the two independent variables are shown in Table 3.

**Table 3:** Experimental design and responses after lyophilization

Run	Factors		Responses						
	% (g/100 mL)		%	Log_10_ CFU/mL					
	S	SM	Y_1_	Y_2_	Y_3_	Y_4_	Y_5_	Y_6_	Y_7_
1	0.00	0.00	50.03	5.00	3.59	2.58	1.58	1.57	1.56
2	6.00	0.00	62.61	11.77	10.70	10.67	10.64	10.59	10.55
3	0.00	6.00	67.63	11.78	10.70	10.68	10.66	10.60	10.56
4	6.00	6.00	76.19	10.79	9.09	9.06	9.00	8.77	8.72
5	− 1.24^a^	3.00	60.21	8.56	7.56	7.53	7.49	7.42	7.39
6	7.24	3.00	68.83	11.05	10.34	10.33	10.30	10.26	10.20
7	3.00	− 1.24^a^	57.62	8.54	7.75	7.52	7.46	7.41	7.37
8	3.00	7.24	74.86	11.33	10.40	10.34	10.32	10.30	10.23
9	3.00	3.00	65.96	10.06	9.19	9.05	8.52	8.50	8.48
10	3.00	3.00	65.73	10.06	9.19	9.05	8.52	8.50	8.48
11	3.00	3.00	65.97	10.06	9.19	9.05	8.52	8.50	8.48
12	3.00	3.00	65.72	10.06	9.19	9.05	8.52	8.50	8.48
13	3.00	3.00	65.45	10.06	9.19	9.05	8.52	8.50	8.48

^a^As negative concentration cannot be achieved 0 was considered

Linear model for survival rate and 2FI model for rest was found to be adequate for the prediction of the response variables. Final equations in terms of coded factors are;

2 $${\text{Y}}_{1} = 65.14 + 4.16*{\text{A}} + 6.94*{\text{B}}$$

3 $${\text{Y}}_{2} = 9.93 + 1.16*{\text{A}} + 1.22*{\text{B}} + - 1.94*{\text{AB}}$$

4 $${\text{Y}}_{3} = 8.93 + 1.18*{\text{A}} + 1.16*{\text{B}} + - 2.18*{\text{AB}}$$

5 $${\text{Y}}_{ 4} = 8. 7 7+ 1. 30*{\text{A}} + 1. 3 1*{\text{B}} + - 2. 4 3*{\text{AB}}$$

6 $${\text{Y}}_{ 5} = 8. 4 7+ 1. 4 2*{\text{A}} + 1. 4 3*{\text{B}} + - 2. 6 8*{\text{AB}}$$

7 $${\text{Y}}_{ 6} = 8. 4 2+ 1. 40*{\text{A + 1}}. 4 1*{\text{B}} + - 2. 7 1*{\text{AB}}$$

8 $${\text{Y}}_{ 7} = 8. 3 8+ 1. 3 9*{\text{A}} + 1. 40*{\text{B}} + - 2. 7 1*{\text{AB}}$$

where Y_1_, Y_2_, Y_3_, Y_4_, Y_5_, Y_6_ and Y_7_ are predicted survival rate (%), log_10_ CFU/mL values obtained in viable count after lyophilization [VCAL], VCAL 1 month, VCAL 6 months, VCAL 12 months, VCAL 18 months and VCAL 24 months, respectively. Similarly, A is the code for sucrose concentration and B for reconstituted skim milk concentration. The regression equations (Eqs. 2–8) of models has been evaluated by using F-test for analysis of variance (ANOVA), which displayed that the regression equations were statistically significant with 95% confidence and prediction intervals (*P* value < 0.05) (Table 4).

**Table 4:** Analysis of variance (ANOVA) of all response variables

Responses	SS	DF	MS	F	P (Pr > F)
Survival rate (%)					
Model	524.74	2	266.37	89.35	< 0.0001**
*A*-*Sucrose*	*138.87*	*1*	*138.87*	*47.29*	< *0.0001*
*B*-*Reconstituted skim milk*	*385.88*	*1*	*385.88*	*131.42*	< *0.0001*
Residual	29.36	10	2.94		
*Lack of fit*	*29.18*	*6*	*4.86*	*106.58*	0.0002*
*Pure error*	*0.18*	*4*	*0.05*		
Cor total	554.11	12			
VCAL					
Model	37.77	3	12.59	92.85	< 0.0001**
*A*-*Sucrose*	*10.81*	*1*	*10.81*	*79.70*	< *0.0001*
*B*-*Reconstituted skim milk*	*11.87*	*1*	*11.87*	*87.58*	< *0.0001*
*AB*	*15.09*	*1*	*15.09*	*111.27*	< *0.0001*
Residual	1.22	9	0.14		
*Lack of fit*	*1.22*	*5*	*0.24*		
*Pure error*	*0.00*	*4*	*0.00*		
Cor total	38.99	12			
VCAL 1 month					
Model	40.80	3	13.60	70.71	< 0.0001**
*A*-*Sucrose*	*11.09*	*1*	*11.09*	*57.68*	< *0.0001*
*B*-*Reconstituted skim milk*	*10.74*	*1*	*10.74*	*55.85*	< *0.0001*
*AB*	*18.96*	*1*	*18.96*	*98.60*	< *0.0001*
Residual	1.73	9	0.19		
*Lack of fit*	*1.73*	*5*	*0.35*		
*Pure error*	*0.00*	*4*	*0.00*		
Cor total	42.53	12			
VCAL 6 months					
Model	50.86	3	16.95	48.39	< 0.0001**
*A*-*Sucrose*	*13.57*	*1*	*13.57*	*38.73*	*0.0002*
*B*-*Reconstituted skim milk*	*13.72*	*1*	*13.72*	*39.17*	*0.0001*
*AB*	*23.57*	*1*	*23.57*	*67.28*	< *0.0001*
Residual	3.15	9	0.35		
*Lack of fit*	*3.15*	*5*	*0.63*		
*Pure error*	*0.00*	*4*	*0.00*		
Cor total	54.02	12			
VCAL 12 months					
Model	61.37	3	20.46	39.64	< 0.0001**
*A*-*Sucrose*	*16.20*	*1*	*16.20*	*31.40*	*0.0003*
*B*-*Reconstituted skim milk*	*16.45*	*1*	*16.45*	*31.88*	*0.0003*
*AB*	*28.72*	*1*	*28.72*	*55.66*	< *0.0001*
Residual	4.64	9	0.52		
*Lack of fit*	*4.64*	*5*	*0.93*		
*Pure error*	*0.00*	*4*	*0.00*		
Cor total	66.02	12			
VCAL 18 months					
Model	61.03	3	20.34	41.44	< 0.0001**
*A*-*Sucrose*	*15.69*	*1*	*15.69*	*31.97*	*0.0003*
*B*-*Reconstituted skim milk*	*15.94*	*1*	*15.94*	*32.47*	*0.0003*
*AB*	*29.39*	*1*	*29.39*	*59.87*	< *0.0001*
Residual	4.42	9	0.49		
*Lack of fit*	*4.42*	*5*	*0.88*		
*Pure error*	*0.00*	*4*	*0.00*		
Cor total	65.45	12			
VCAL 24 months					
Model	60.57	3	20.19	41.68	< 0.0001**
*A*-*Sucrose*	*15.48*	*1*	*15.48*	*31.95*	*0.0003*
*B*-*Reconstituted skim milk*	*15.73*	*1*	*15.73*	*32.48*	*0.0003*
*AB*	*29.36*	*1*	*29.36*	*60.62*	< *0.0001*
Residual	4.36	9	0.48		
*Lack of fit*	*4.36*	*5*	*0.87*		
*Pure error*	*0.00*	*4*	*0.00*		
Cor total	64.93	12			

*DF* degrees of freedom, *SS* sum of squares, *MS* mean square, *F and P (Pr > F)* F and P values, respectively

** *P* < 0.0001, very significant; * *P* < 0.005, significant

**Fig. 1:**
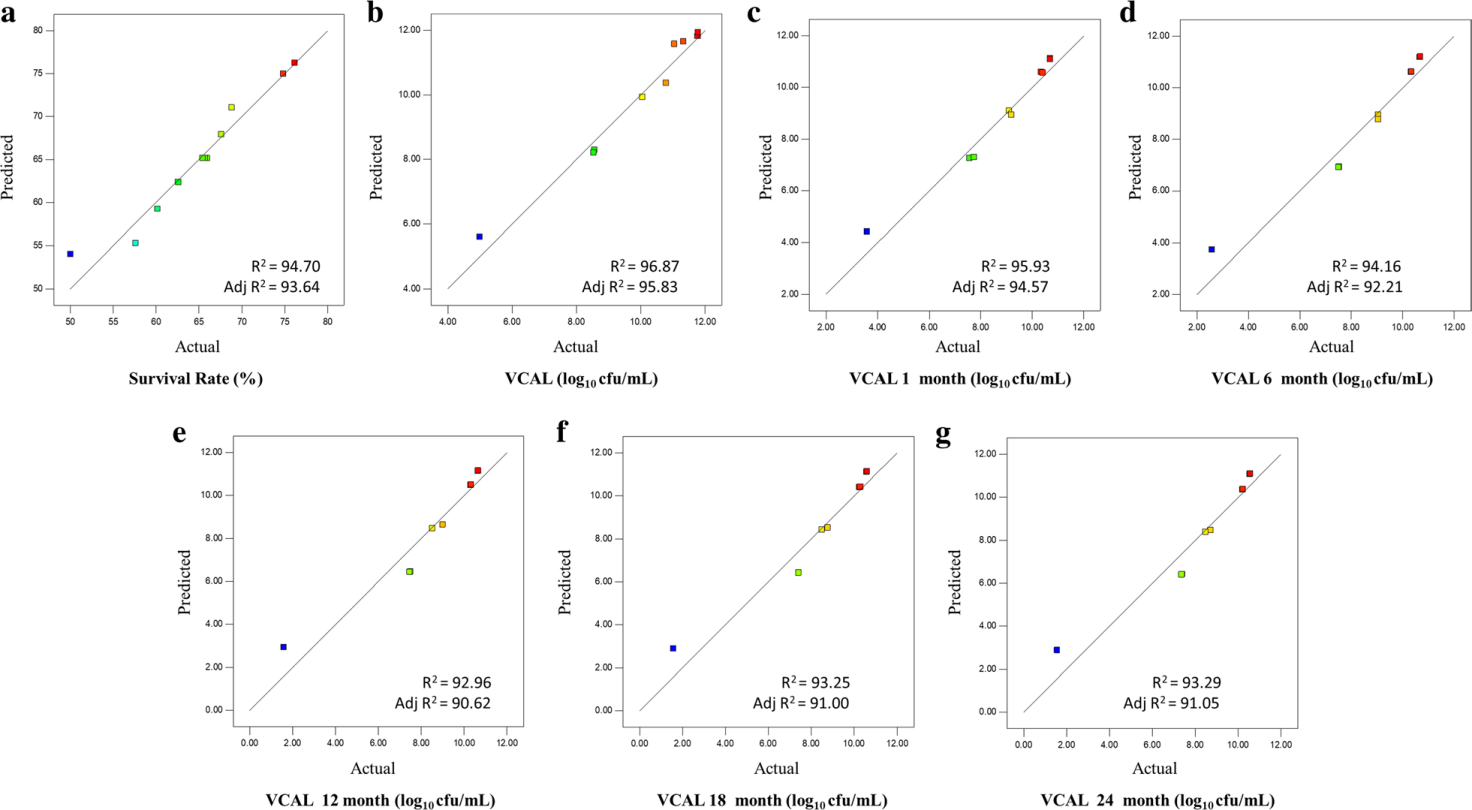
Predicted vs. actual plot of: **a** survival rate (%), log_10_ CFU/mL values obtained in **b** viable count after lyophilization [VCAL], **c** VCAL 1 month, **d** VCAL 6 months, **e** VCAL 12 months, **f** VCAL 18 months and **g** VCAL 24 months lyophilized probiotics powder

Predicted v/s actual plot of fitted models, survival rate (%), log_10_ CFU/mL values obtained in viable count after lyophilization [VCAL], VCAL 1 month, VCAL 6 months, VCAL 12 months, VCAL 18 months and VCAL 24 months has been tabulated in Fig. 1, respectively.

The values of coefficient of determination (R^2^) used to express the ‘fitness’ of the model’s regression equations (Eqs. 2–8) are 0.947 for Y_1_, 0.969 for Y_2_, 0.959 for Y_3_, 0.942 for Y_4_, 0.929 for Y_5_, 0.932 for Y_6_, 0.933 for Y_7_ which further pointed out that Eqs. (2)–(8) may be utilized for representing the true relationships among the variables. Since the calculated values of R^2^ and adjusted-R^2^ vary negligibly, models are composed of significant terms. An adequate precision ratio was determined by the software highlighting the signal (information of interest) to noise (random errors) ratio arising out of the data set. Adequate precision ratio greater than 4 is desirable and its values in the current investigation was 26.9 for survival rate, 30.9 for VCAL, 27.6 for VCAL 1 month, 22.7 for VCAL 6 months, 20.6 for VCAL 12 months, 21.2 for VCAL 18 months and 21.3 for VCAL 24 months, respectively. The above value shows that the originating model can be used to navigate in the design space. Further, low coefficient of variation (CV) values (2.63 for survival rate, 3.71 for VCAL, 4.91 for VCAL 1 month, 6.75 for VCAL 6 months, 8.49 for VCAL 12 months, 8.32 for VCAL 18 months and 8.3 for VCAL 24 months) showed the precision and reliability of the experiments. Above results indicated that these models will be helpful in further optimization by CCRD.

Low P-values (< 0.05) of each model term indicated the significant effect of factor on results. As per the P-values tabulated in Table 4, A (sucrose) and B (reconstituted skim milk) were showing the significance in the survival rate model. Further, in remaining models the significant terms were A (sucrose), B (reconstituted skim milk) and interaction variable AB (sucrose × reconstituted skim milk). The responses levels were decreasing with increase in the levels of factors as indicated by negative sign in Eqs. (2)–(8). The graphical representation of results in 2D contour plots and 3D surface plots of the regression model indicated the interactions between model terms A and B (Ramakrishnan et al. 2012, 2013).

**Fig. 2:**
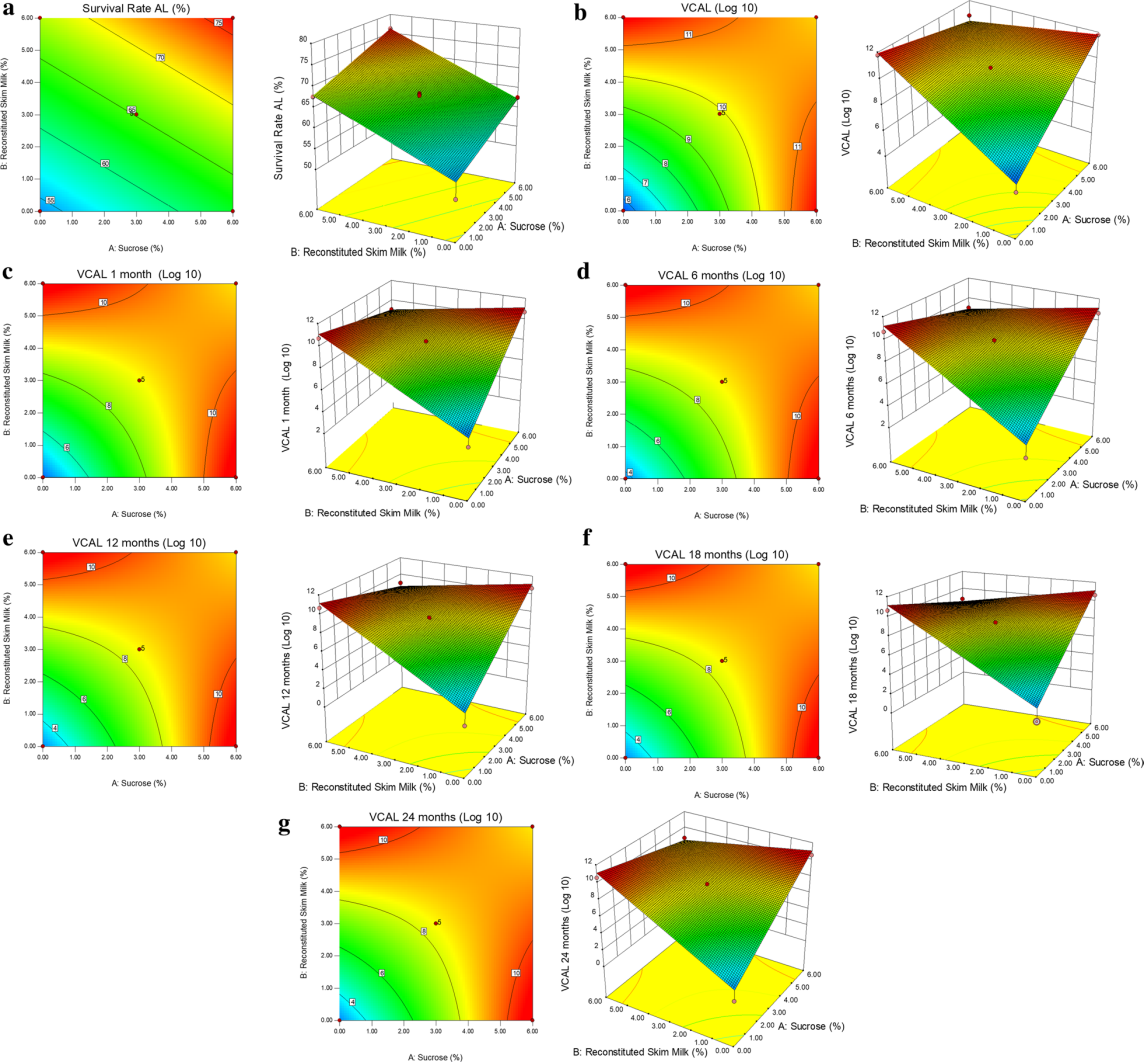
Response surface (right) and contour (left) plots depicting effect of sucrose and reconstituted skim milk on **a** survival rate (%), log_10_ CFU/mL values obtained in **b** viable count after lyophilization [VCAL], **c** VCAL 1 month, **d** VCAL 6 months, **e** VCAL 12 months, **f** VCAL 18 months and **g** VCAL 24 months lyophilized probiotics powder

The least count was found at the zero concentration of sucrose and reconstituted skim milk in all regression models (Fig. 2). The results stressed upon the exclusive use of sucrose and reconstituted skim milk without altering any of the culture conditions for having higher viable count. In addition, the viable count increased when maximum concentration of sucrose (6%) and reconstituted skim milk (6%) were used, individually. The viable count decreased when highest (at 6%) concentrations of sucrose and reconstituted skim milk used together. The measure of concentrations of sucrose and reconstituted skim milk was crucial for viable count of *L. acidophilus* as represented in Fig. 2.

**Fig. 3:**
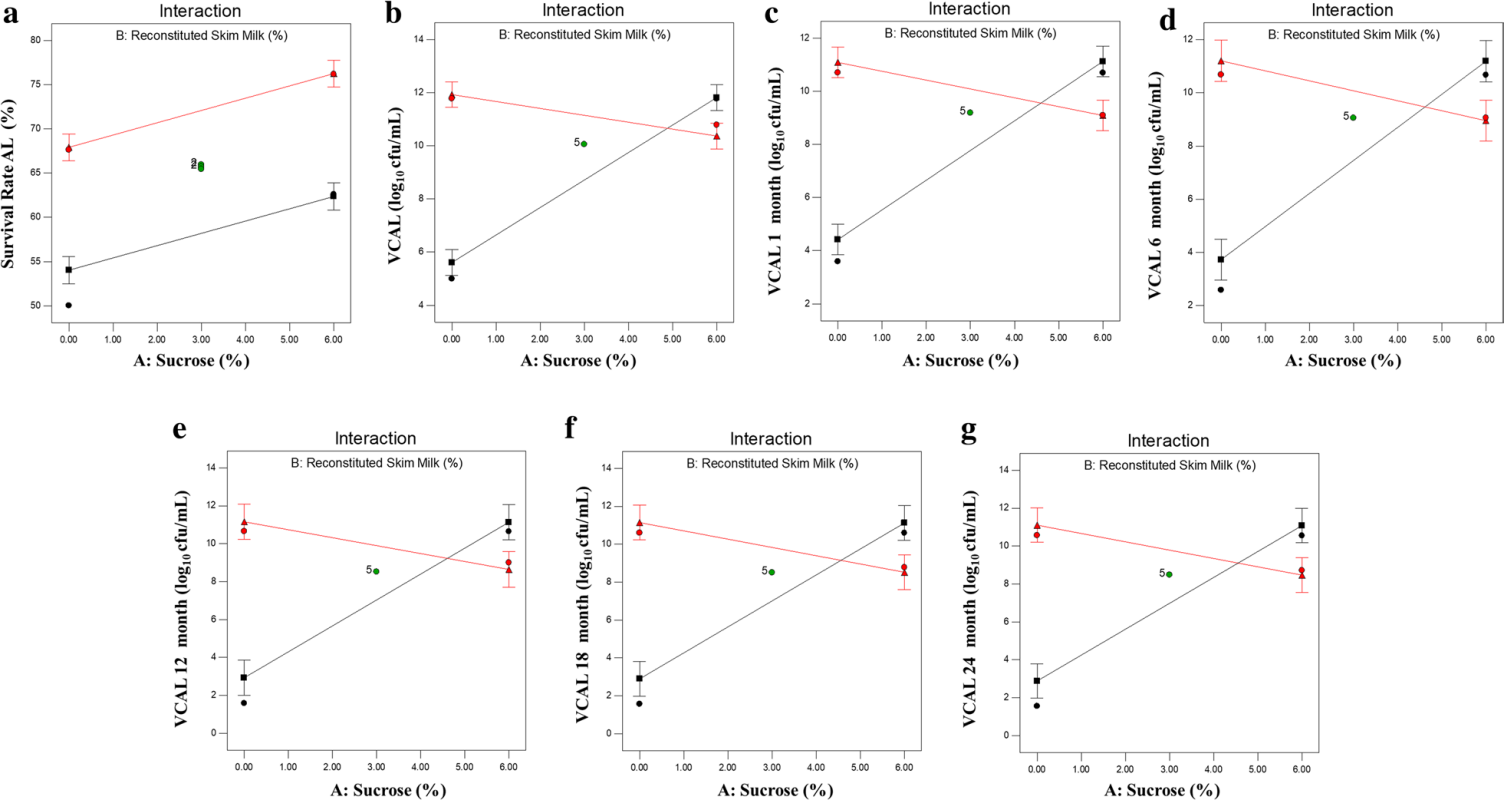
Interaction of sucrose versus reconstituted skim milk on **a** Survival Rate (%), log10 CFU/mL values obtained in **b** Viable count after lyophilization[VCAL], **c** VCAL 1 month, **d** VCAL 6 months, **e** VCAL 12 months, **f** VCAL 18 months and **g **VCAL 24 months lyophilized probiotics powder

Figure 3 depicts an interaction plot of the model; where we noticed in Fig. 3b–g that when we used low concentrations of sucrose and reconstituted skim milk, the viability was low. Whereas, with increased concentration of sucrose (6%) and minimal concentration of reconstituted skim milk; we could see increase in viable count but decrease in survival rate. We further noticed that when we used low sucrose concentration with high concentration of reconstituted skim milk, the viability was high and with increasing concentration of both sucrose and reconstituted skim milk above ~ 5%, the viability remains high regardless of reconstituted skim milk concentration. The Least Significant Difference (LSD) bars overlap at this end of the interaction graph, which implies that there is no significant difference in viability. The effect on survival rate due to the interaction of sucrose as well as reconstituted skim milk is presented in Fig. 3a. Figure 3b–g are indicative of interaction of two-factor shown by two characteristic non-parallel lines. The non-overlapping LSD bars indicated the significant effect of sucrose.

**Fig. 4:**
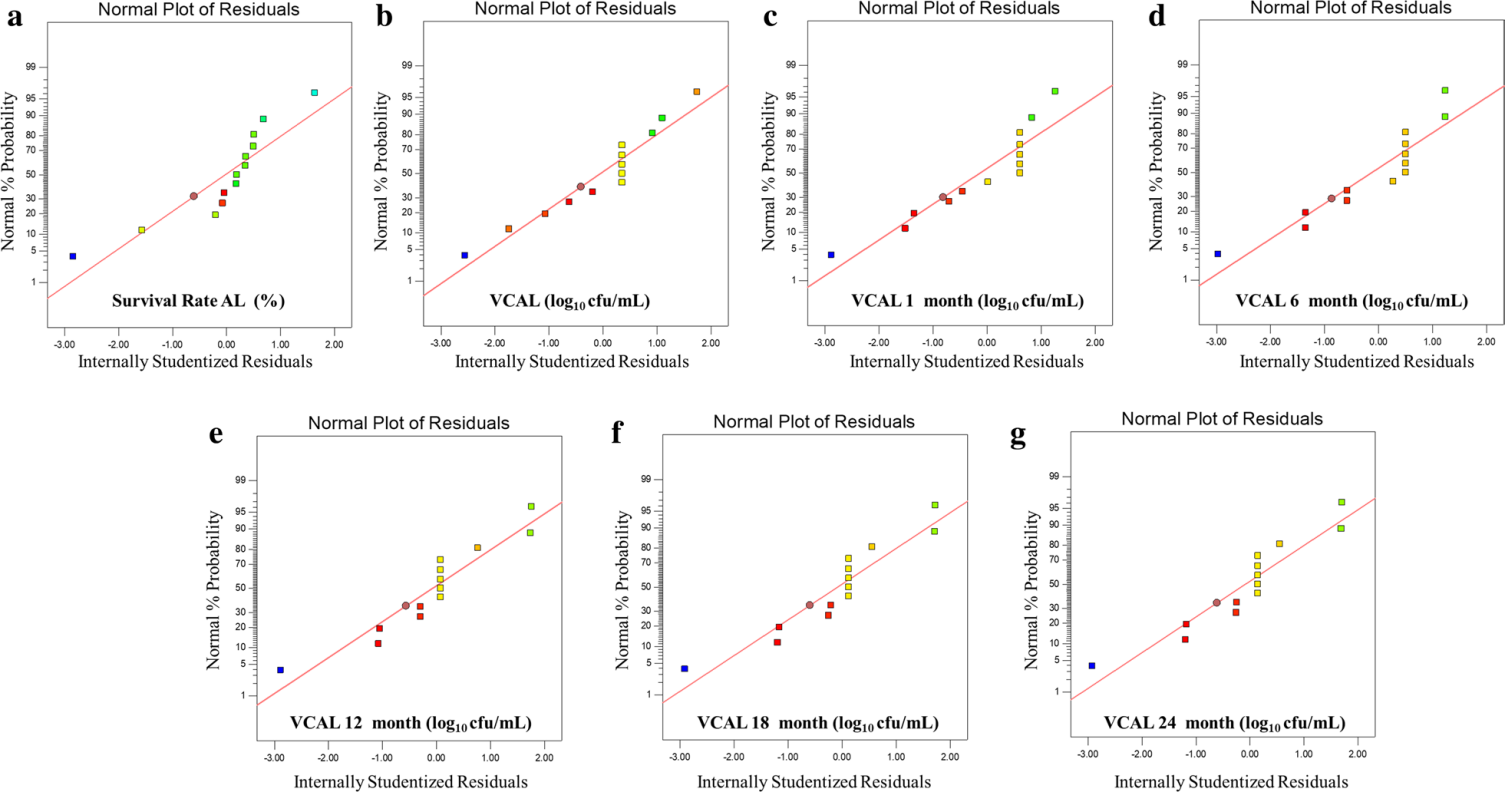
Normal plot of residuals for responses **a** survival rate (%), log_10_ CFU/mL values obtained in **b** viable count after lyophilization [VCAL], **c** VCAL 1 month, **d** VCAL 6 months, **e** VCAL 12 months, **f** VCAL 18 months and **g** VCAL 24 months lyophilized probiotics powder

During diagnostics, we noticed through the normal plot for the residuals (Fig. 4), that the residuals for all responses exhibit no major deviations from the linearity showing that the residuals are normally distributed. This validated the statistical assumptions of the model.

### Optimization

**Fig. 5:**
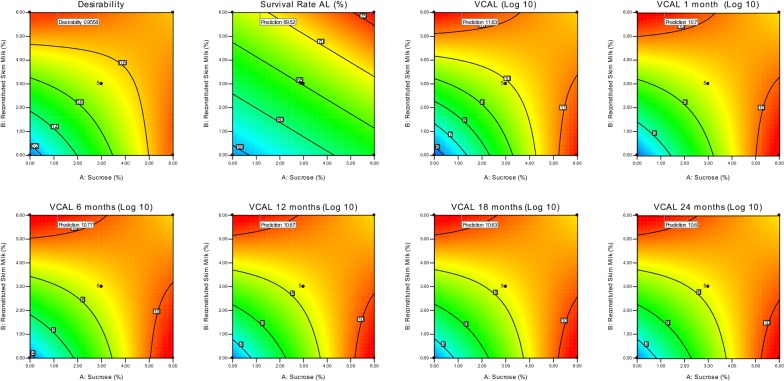
Contour plots depicting the predicted optimized response values

In order to optimize the multiple responses for achieving the desired point in the design region, we used the simultaneous optimization by RSM. The motive of the present investigation was to maximize *L. acidophilus* survival rate during lyophilization and storage. Design-Expert^®^ software was employed for numerical optimization and seven solutions were suggested by the software. Out of which, we selected the default first solution that predicted the maximum response upon use of recommend ratio of sucrose 1.16% and reconstituted skim milk 6.0% in suspension medium (Fig. 5). The responses for NCDC 291, NCDC 015 and MTCC 10307 strains upon optimization are tabulated in Table 5. Confirmation test value results shows that there is a close agreement with predicted values at a 95% confidence and prediction interval establishing the models validity.

**Table 5:** Optimum process and validation experiment results at 95% confidence and prediction interval

Responses	Target	Predicted results	Confirmation test results for NCDC 291	Confirmation test results for NCDC 015	Confirmation test results for MTCC 10307	95% CI low	95% CI high	95% PI low	95% PI high
Survival rate AL	Maximize	69.52	69.86	68.84	67.92	67.66	71.39	65.35	73.70
VCAL	Maximize	11.63	11.33	11.04	11.05	11.14	12.12	10.66	12.59
VCAL 1 month	Maximize	10.70	10.50	10.43	10.42	10.12	11.28	9.55	11.85
VCAL 6 months	Maximize	10.77	10.45	10.39	10.38	9.98	11.55	9.22	12.32
VCAL 12 months	Maximize	10.67	10.44	10.38	10.37	9.72	11.62	8.79	12.56
VCAL 18 months	Maximize	10.63	10.43	10.38	10.36	9.71	11.56	8.80	12.47
VCAL 24 months	Maximize	10.60	10.42	10.37	10.34	9.67	11.52	8.77	12.42

## Discussion

In this work, we have optimized the lyophilization and storage conditions of 3 known *L. acidophilus* strains with proven commercial usage profile as a probiotic supplement. As reported by earlier researchers, the viability of probiotic cultures after lyophilization and storage depends on the several factors (Broeckx et al. 2016; Kailasapathy 2013). From our preliminary investigations, we have optimized the pre-lyophilization factors like inoculum size, culture phase and revival factors such as temperature, volume and time which are in agreement to previous studies (Montel Mendoza et al. 2014). In addition, we further examined the effect of lyoprotectors on viability and storage of post-lyophilized cultures.

We initiated our work with two lyoprotectors in the culture media composing of skim milk and sucrose in a cost-effective manner for better solubility of lyophilized powder as suggested by other researchers (Jalali et al. 2012). The present investigation revealed the dependency of both strain and lyoprotector on the survivability of *L. acidophilus* during lyophilization and storage. The tested lyoprotectors were effective in protecting the cells during the lyophilization process. After optimization, it appears at first instance the Y2–Y7 response values obtained for runs 1 and 2 (Table 3) remain more constant than optimized value (Table 5). On closer inspection, the overall survival rate of the cultures on optimization was far better than the test runs even after several repetitions. The superior performance was obtained with minimal concentration (~ 1.2%) of sucrose suspended in skim milk as compared to other formulations (Table 5). The use of RSM-CCRD in evaluating survival behavior of lactobacilli during storage resulted into viability plots that fit well with the applied model. From the application of statistical analysis, it confirms the effect of variable lyoprotectors on the estimated survival parameters of each individual strain among the three used.

It is evident from our investigation that addition of skim milk enhanced the survival rate with improved resistance to lyophilization stress in the presence of sucrose (Figs. 2, 3). The results obtained during the lyophilization process were consistent with earlier agreements indicating skim milk alone was not a good lyoprotectant during lyophilization of lactic acid bacteria (Otero et al. 2007). Similar studies have suggested the significant enhancement of protective abilities on addition of lyoprotectors in a mixture composed of skim milk, lactose, and sodium ascorbate during lyophilization (Chen et al. 2015).

Importantly, the storage conditions on the recovery of lyophilized cells were found to be critical for their survival (Goderska 2012). Also, it is clearly evident by the 2-year study that the lyophilized cells in a tightly wrapped cryovials placed in an airtight container and stored in dark at 4 °C was enough for the cultures to be viable. In addition, the stored cryovials containing lyophilized cells were able to resuscitate in rehydrating medium, more efficiently. The resultant data is indicative that microorganism’s resistance against lyophilization and storage found to be highly variable and strain-dependent. Certain lyoprotectors have bearing among themselves in protecting cells throughout lyophilization and storage. The current work is being considered to link with the formulations of symbiotic macrodevices [SynMacDev] for eubiosis of gut microflora, is underway.

In summary, optimization of lyoprotectants during lyophilization and long-term storage of *L. acidophilus* was investigated using statistical modelling. The RSM-CCRD was used for statistical analysis and optimization of the process. The concentration of sucrose and reconstituted skim milk had a potential bearing on the survival of *L. acidophilus* was evaluated over a period of 2 years. The most conspicuous result to emerge from the data is that the suspension medium composed of minimal concentration of sucrose (~ 1.2%) and reconstituted skim milk (6%) was found to be significantly effective in maintaining high degree of survival even after lyophilization and stable for longer storage at 4 °C. The developed regimen substantiates the necessity of the preadaptation of the individual strains to gastrointestinal conditions by mimicking the environmental factors include temperature and pH. From this investigation, the optimized suspension media used to obtain the lyophilized concentrate of *L. acidophilus* could be recommended in designing dietary supplement with minimal modification for industrial application. Thus reducing the manufacturing cost as well as storage loss occurring both in pharma and food sector.

## Additional file

**Additional file 1.** Design Summary & Evaluation with additional tables and figure.

## Abbreviations

CFU: colony forming unit
CCRD: central composite rotatable design
RSM: response surface methodology
2FI: two factorial interaction
SM: reconstituted skim milk
S: sucrose
VCAL: viable count after lyophilization
LSD: least significant difference
