# Supplementary material for: Statistical modelling for optimized lyophilization of Lactobacillus acidophilus strains for improved viability and stability using response surface methodology
Source: AMB Express. 2018 Aug 10;8:129. doi: 10.1186/s13568-018-0659-3 (PMC6086920; doi:10.1186/s13568-018-0659-3)
Supplement: Supplementary file 1 — Additional file 1. Design Summary & Evaluation with additional tables and figure. [file 13568_2018_659_MOESM1_ESM.pdf]

AMB Express

Statistical modelling for optimized lyophilization of *Lactobacillus acidophilus* strains for improved viability and stability using response surface methodology.

LAKSHMINARAYANA T.S.[1] and BASAVARAJ MADHUSUDHAN[1,2]*

[1] Research Center *for* Nanoscience and Technology, Department of Studies and Research in Biochemistry, Bio-Science Block, Davangere University, Shivagangotri, Davanagere-577002, Karnataka, India

[2] Research Center *for* Nanoscience and Technology, Department of Studies and Research in Food Technology, Bio-Science Block, Davangere University, Shivagangotri, Davanagere-577002, Karnataka, India.

* Corresponding author

E-mail : madhusudhanbasavaraj@gmail.com

Address : Research Center *for* Nanoscience and Technology, Department of Studies and Research in Biochemistry & Food Technology, Bio-Science Block, Davangere University, Shivagangotri, Davanagere-577002, Karnataka, India.

ORCID ID : 0000-0002-9647-7019 (Basavaraj Madhusudhan)

ORCID ID : 0000-0002-3419-2640 (Lakshminarayana T.S)

The following information is provided for the sake of clarity, transparency in details of study carried out.

Table S1. Design Summary

**Study Type**	Response Surface	**Runs**	13
**Initial Design**	Central Composite	**Blocks**	No Blocks

Design Model Quadratic

Factor	Name	Units	Type	Low Actual	High Actual	Low Coded	High Coded	Mean	Std. Dev.
A	Sucrose	%	Numeric	0.00	6.00	-1.00	1.00	3.00	2.353
B	Reconstituted Skim Milk	%	Numeric	0.00	6.00	-1.00	1.00	3.00	2.353

Response	Name	Units	Obs	Analysis	Minimum	Maximum	Mean	Std. Dev.	Ratio	Trans	Model
R1	Survival Rate AL	%	13	Polynomial	50.03	76.19	65.14	6.53	1.52	None	Linear
R2	VCAL	Log 10	13	Polynomial	5.00	11.78	9.93	1.73	2.36	None	2FI
R3	VCAL 1 month	Log 10	13	Polynomial	3.59	10.70	8.93	1.81	2.98	None	2FI
R4	VCAL 6 months	Log 10	13	Polynomial	2.58	10.68	8.77	2.04	4.13	None	2FI
R5	VCAL 12 months	Log 10	13	Polynomial	1.58	10.66	8.47	2.25	6.74	None	2FI
R6	VCAL 18 months	Log 10	13	Polynomial	1.57	10.60	8.42	2.24	6.76	None	2FI
R7	VCAL 24 months	Log 10	13	Polynomial	1.56	10.56	8.38	2.23	6.79	None	2FI

Table S2. Design Evaluation

Degrees of Freedom for Evaluation	
Model	5
Residuals	7
Lack 0f Fit	3
Pure Error	4
Corr Total	12

				Power at 5 % alpha level for effect of		
**Term**	**StdErr****	**VIF**	**Ri-Squared**	**0.5 Std. Dev.**	**1 Std. Dev.**	**2 Std. Dev.**
A	0.35	1.00	0.0000	9.4 %	23.2 %	68.1 %
B	0.35	1.00	0.0000	9.4 %	23.2 %	68.1 %
AB	0.50	1.00	0.0000	7.2 %	14.0 %	40.8 %
A^2	0.38	1.02	0.0170	20.8 %	62.1 %	99.4 %
B^2	0.38	1.02	0.0170	20.8%	62.1 %	99.4 %

****Basis Std. Dev. = 1.0**

Measures Derived From the $(X'X)^{-1}$ Matrix

Std	Leverage	Point Type
1	0.6250	Fact
2	0.6250	Fact
3	0.6250	Fact
4	0.6250	Fact
5	0.6250	Axial
6	0.6250	Axial
7	0.6250	Axial
8	0.6250	Axial
9	0.2000	Center
10	0.2000	Center
11	0.2000	Center
12	0.2000	Center
13	0.2000	Center
**Average =**	**0.4615**	

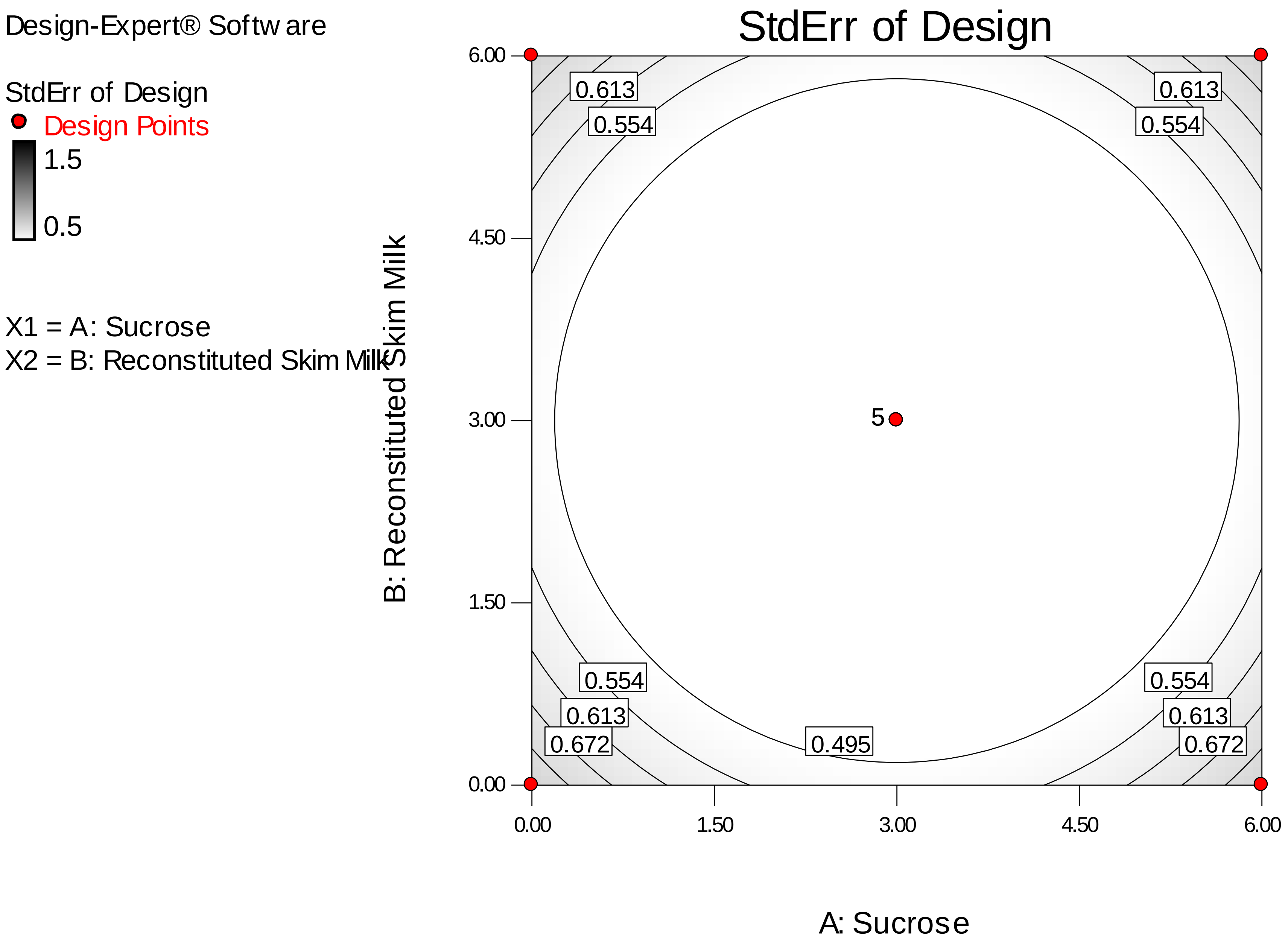

Figure S1. Standard Error of Design.
